# Public involvement in an aggregate and individual participant data meta-analysis of mindfulness-based programmes for mental health promotion

**DOI:** 10.1186/s13643-024-02601-5

**Published:** 2024-08-06

**Authors:** Claire Friedrich, Toni Fairbairn, Graham Denton, Mary Geddes, Darren Thomas-Carr, Peter B. Jones, Julieta Galante

**Affiliations:** 1 Department of Psychiatry, University of Cambridge, Douglas House, 18B Trumpington Road, Cambridge, CB2 8AH UK; 2 Department of Psychiatry, University of Oxford, Oxford, UK; 3 Cambridge, UK; 4 National Institute for Health Research (NIHR) Applied Research Collaboration East of England, Cambridge, UK; 5 Contemplative Studies Centre, Melbourne School of Psychological Sciences, Faculty of Medicine, Dentistry, and Health Sciences, University of Melbourne, Melbourne, Australia

**Keywords:** Patient and public involvement, Stakeholder involvement, Systematic review, Individual participant data meta-analysis, Mindfulness

## Abstract

**Background:**

Involving the public in evidence synthesis research is challenging due to the highly analytic nature of the projects, so it is important that involvement processes are documented, reflected upon, and shared to devise best practices. There is a literature gap on the involvement of the public in individual participant data meta-analyses, particularly in public health projects. We aimed to document and reflect on our collective experiences of involving and being involved as public stakeholders at all stages of a systematic review and individual participant data meta-analysis project.

**Methods:**

We formed a stakeholder group made of four members of the public at the beginning of our evidence synthesis project comprising a systematic review, an aggregate data meta-analysis, and an individual participant data meta-analysis of mindfulness-based programmes for mental health promotion in non-clinical adults. Following each group meeting, members and participating researchers completed written reflections; one group member collected and collated these. At the end of the project, a reflective writing workshop was held before all members completed their final reflections. Everyone completed an adapted, open-ended questionnaire which asked about what did and did not work well, the overall experience, what could be improved, and the felt impact the stakeholder group had on the research.

**Results:**

Overall, the stakeholders and researchers reported a positive experience of working together. Positives from the stakeholders’ point of view included learning new skills, experiencing research, and making new friends. For the researchers, stakeholders helped them focus on what matters to the public and were reinvigorating research partners. The challenges stakeholders experienced included having long gaps between meetings and feeling overwhelmed. The researchers found it challenging to strike the balance between asking stakeholders to be involved and for them to learn research-related skills without overburdening them and making sure that the learning was engaging. When looking back at their experience, stakeholders described seeing their impact on the project in hindsight but that this was not felt while the project was being carried out.

**Conclusion:**

Successfully involving the public in complex evidence synthesis projects is possible and valuable from the points of view of the researchers and the stakeholders. However, it requires a significant time, skill, and resource investment that needs to be factored in from project inception. Further guidance and stakeholder training materials would be helpful. Specific suggestions are provided.

## Background

Patient and public involvement (PPI) in health research describes involving individuals with user knowledge and lived experience of the research topic who do not primarily carry out the research [1]. Non-profit organisation INVOLVE defines public involvement as research that is carried out by or with members of the public rather than about or for them [2]. PPI group members (from now on ‘stakeholders’) can include professionals, patients and caregivers, or the public, although the latter is less common. Stakeholders are invited to bring lived experiences to the research with the goal of improving the relevancy of research aims and quality [3]. Benefits of stakeholder involvement include prioritising research questions, selecting more relevant outcomes, creating innovative solutions and new ideas, and ensuring equality of public access to policy and decision-making [3]. It is becoming more common practice to involve stakeholders in health research in the UK, and stakeholder involvement is now fundamental to government-funded health research [4]. Stakeholders can be invited to join research projects of any methodologies, including intervention trials, qualitative research, or evidence syntheses such as systematic reviews (SRs) [5]. Stakeholders will engage in a wide range of research activities in keeping with the project aims and methodologies.

When involved in intervention studies, they may support guiding the intervention design, recruiting participants, or selecting outcomes [5]. However, engaging the public in evidence synthesis projects is more challenging because there are no interventions to design and no participants to recruit; instead, most of the processes tend to be quite technical, requiring specific research skills. Still, stakeholders engaged in SR projects have contributed by finalising research questions and protocols, selecting outcomes, making sense of findings, and supporting dissemination tasks [6, 7]. The benefits of stakeholder involvement in SRs include increasing the relevancy of the research, providing a real-world context, and enhancing end-user uptake of the interventions or treatments reviewed [8]. Involving stakeholders can improve the actual and perceived usefulness of SRs and is likely to reduce research waste [9].

Researchers are incorporating stakeholder groups within their projects at increasing rates [10], leading to clearer recommendations around involving stakeholder members and for reporting on and assessing the impact of their involvement [10]. However, evidence and guidance are limited specifically within the context of evidence synthesis projects and when involving public, less so professional or patient, stakeholders [1]. Additionally, the level and clarity of reporting are inconsistent with reviews not reporting stakeholder characteristics or their involvement activities, and none measured the impact of involving stakeholders [1].

There are developments around stakeholder impact guidance and frameworks, such as the Public Involvement Impact Assessment Framework [11] and the Guidance for Reporting Involvement of Patients and the Public (GRIPP2) checklist [12, 13], however a lack of information on actual stakeholder involvement throughout the SR process remains [9]. In response to this gap, the ACTIVE framework (Authors and Consumers Together Impacting on Evidence) has been developed and can be accessed as online training via Cochrane [14]. It covers the essentials of good practice such as defining stakeholder roles and responsibilities and forming good relationships between researchers and stakeholders through respect, trust, confidentiality, and clear communication [9]. These recommendations are in keeping with the NIHR INVOLVE values and principles framework [2] which Greenhalgh et al. [15] use as an example of a partnership-focused framework due to its standards set around inclusive opportunities and working together in respectful, supportive, and transparent ways.

Greenhalgh et al. [15] synthesised 65 stakeholder involvement frameworks and report large heterogeneity and limited transferability across these frameworks indicating that a ‘one-size-fits-all’ framework may not be suitable for guiding researchers in their design and implementation of stakeholder involvement. Instead, they suggest making evidence-based resources available for researchers to co-design their own framework with project stakeholders [15], using a co-design approach could certainly make the framework more applicable to individual project and allow for more input from stakeholders. If using an approach without established frameworks, researchers should have access to reports on stakeholder and researcher experiences to support their decision-making and co-design of their own framework.

Due to the limited evidence and reporting of stakeholder experiences in evidence synthesis work, it is recommended that researchers carrying out evidence synthesis projects collect data on the stakeholder experience from the stakeholders themselves [9]. Not many reviews have described the experience of researchers, and even less so of the stakeholders themselves, when involving stakeholders in evidence synthesis projects. In one qualitative meta-synthesis project, stakeholders received training, helped develop a coding frame and attended a focus group to support the researchers in interpreting the data [7]. Stakeholders provided mixed feedback on their ability to understand research and medical terminology throughout the project and in the training material; they wanted face-to-face training and feedback from the researchers to know how they were doing [7]. Stakeholders gave positive feedback and happily be involved in future projects, although questioned the benefit and extent of their contribution [7], which is similar to the feedback collected by Vale et al. [16].

In an individual participant data meta-analysis (IPD-MA) patient stakeholders, named ‘Patient Research Partners’ were involved in recruiting professional stakeholders, providing feedback on documents, contributing to project newsletters, and providing input into the lay summary for the Cochrane review [16]. The group in general had a positive experience and all would do it again; however, as with Bayliss et al. [7], some questioned the usefulness of their input in the context of a SR as the outcomes are pre-set to what trials had already collected [16].

It is necessary to capture feedback from stakeholders, because reviews which do not will miss out on a richer level of information and are not able to provide guidance for future researchers to help them with planning their training material and method of stakeholder involvement. This paper examines the experience of public stakeholders within a 4-year long, two-stage SR project looking at mindfulness-based interventions for mental health promotion in a non-clinical sample. The findings of each stage are published elsewhere [17, 18]. The first stage involved completing a SR and aggregate data meta-analysis (AD-MA) of 136 randomised controlled trials (RCTs) of mindfulness-based interventions for mental health promotion in community adults [17]. Then we completed an IPD-MA with the data of 13 RCTs with the same intervention and population types [18, 19]. Public stakeholders were involved at all stages, including in co-authoring this paper.

Following the involvement of a stakeholder group in their SR and IPD-MA project, Vale et al. [16] encourage future researchers to report on the involvement and experiences of stakeholders in their reviews. While Bayliss et al. [7] recommend further research to monitor and evaluate stakeholder involvement, both of which we intend to do. We aim to address the current lack of evidence and guidance of public stakeholder involvement in evidence synthesis projects by documenting and analysing reflections of our collective experiences of involving and being involved as stakeholder members. Researchers and stakeholders co-author this paper to fully understand their experience and to help build evidence around what might be helpful or unhelpful in involving stakeholders in evidence synthesis projects. As the stakeholder group joined a project carrying out specifically an AD-MA and IPD-MA, this reflective work is carried out within the context of these particular types of MAs. To our knowledge, this is the first paper to examine the experience of non-clinical stakeholders’ involvement in an IPD-MA project.

## Methods

### Forming the stakeholder group

Members of the public were invited to join researchers within the Department of Psychiatry at the University of Cambridge. The PI (JG) looked to recruit people with diverse experiences and interests in mental health promotion and mindfulness. Potential stakeholder members were recruited through personal networks and social media advertisements in Spring 2018.

As the SRs conducted examined non-clinical adult samples, the stakeholders were not recruited for having any particular clinical characteristics. However, there was a requirement to achieve diversity in terms of gender, age (aside from having to be adults over 18 years old), and experience with mindfulness practices. Therefore, stakeholders were carefully chosen. In addition, due to the requirement to hold in-person meetings in university buildings in Cambridge, recruited stakeholders had to be local to the Cambridgeshire area. They were given information by the PI about the study and expectations, such as anticipated time commitment, and had the opportunity to discuss the role in detail before committing.

The resulting stakeholder group was made up of two men and two women, ages ranged from 40 to 60s, with varied professional and educational backgrounds. One member had previously attended a Mindfulness-Based Stress Reduction (MBSR) course, one member had a long-term mindfulness meditation practice, while another member had no mindfulness experience (yet experienced in other contemplative practices), and finally, one member had had a dissatisfying initial engagement with mindfulness meditation but was very interested in mental health promotion.

Professional stakeholders were additionally recruited to form an advisory group with the help of public stakeholders. Professional stakeholders ranged in professional background and in terms of personal experiences and opinions of mindfulness. They were kept up to date and their input was sought throughout the study; however, this paper focuses on the experience of public stakeholders so unless specified otherwise, the use of the term stakeholders remains in reference to our public stakeholder group.

### Running the stakeholder group

Stakeholders were involved from the project’s outset. Regular meetings were organised by the PI throughout: two workshops and ten group meetings took place in total from June 2018 to July 2022. In-person meetings lasted between 2 and 4 h. From March 2020, meetings shifted online in response to Covid-19 (with shorter durations) and were once again, conducted in-person from October 2021 onwards. Members were able to attend most meetings and workshops and were reimbursed for their time and transport immediately after attending.

As members were new to health research, about half of the time in initial meetings was used for induction by the PI, covering research background and project aims, timelines, and analyses and explaining the stakeholder role. The training also covered research methodology, specifically RCTs and SRs with MAs. The PI put together an induction document explaining research background, and a glossary. She also encouraged stakeholders to complete Cochrane Collaboration training on SRs for stakeholders [20]. Later, meetings were used to provide study updates and results, and stakeholders were encouraged to provide feedback. Two workshops relating to effective communication (suggested by the PI) and reflective writing (collectively decided) were organised with external workshop leaders in which researchers and stakeholders were trained together. Members of the group also acted as research partners throughout and took part in different SR and dissemination tasks.

### Stakeholders as research partners

Stakeholders were involved in the SR process after receiving training from the PI and a research assistant. Two members were involved in the title and abstract screening using Covidence [21]. Then, following further training, an additional member joined for the data extraction stage, again using Covidence.

After publication of the SR and AD-MA [17], all members stayed on as stakeholders while the IPD-MA was carried out and published [18]. Stakeholders also participated in co-producing a project film, where the review process was summarised, and findings were disseminated. They were involved in the planning and scriptwriting, as well as the acting and voice-over. Their final stakeholder task was to provide reflections on their experiences as stakeholders and to complete this reflective paper together with the researchers. One stakeholder (TF) oversaw collecting the post-meeting reflections and final evaluation questionnaire and analysed these for this paper.

### Stakeholder and researcher reflections

Throughout the project, stakeholders and researchers completed short, personal reflections following each meeting and submitted these to the stakeholder in charge per email. These reflections were to outline their thoughts and experiences in their project involvement and to later write this reflective paper on their experiences. Additionally, nearing the end of the project, a reflective writing workshop was organised as stakeholders and researchers expressed a need for it. Following the workshop, stakeholders and researchers completed an adapted version of the Stakeholders Evaluation Questionnaire designed and shared with us by Vale et al. [16]. The questionnaire was adapted with the help of one stakeholder (TF), and questions covered a range of experiences, asking about their motivation for involvement as a stakeholder, the quality of information and training they received, and their thoughts on their level of involvement. 

Additionally, they were asked about the stakeholder group itself, their thoughts on the group size and diversity, the length and frequency of meetings, and their expectations at the project start and if these were met. Reflective questions asked about what the best and worst aspects of being involved in the project were, what their felt impact on the research was, and whether they would do it again or recommend getting involved in SR and MA projects to other people. Suggestions for improving future stakeholder recruitment, training, and involvement were also sought. Members were repeatedly reminded to be honest in their responses and that the researchers would not be upset by negative feedback. A summary of stakeholders’ and researchers’ experience of stakeholder involvement in the project was established through combining the questionnaire responses and post-meeting feedback. One stakeholder (TF) and the researchers gathered individual questionnaire answers and then grouped together responses into emerging themes guided by the existing questionnaire items and stages of stakeholder involvement (e.g., experience at the start of being involved and reflections looking back over their involvement when the project was completed). The emergent themes and selected quotes to support these were finalised through wider group and PI feedback.

## Results

All four stakeholders and the PI completed the reflections questionnaire in October 2022. Stakeholders, the PI, and researcher also completed reflections following group meetings and workshops throughout the course of the project. On average, group members completed four post-meeting reflections, and everyone completed the Stakeholder Evaluation Questionnaire [16].

### Information provided and level of understanding among stakeholders

Stakeholders reported that, despite being unfamiliar with the research process and methodologies, the PI’s explanations about key research concepts and the use of simple but interactive exercises made their involvement a positive experience. They felt that alongside the well-balanced explanations and background information provided by the researcher, frequent breaks and opportunities to ask questions made it more stimulating and enjoyable.

Three of the four group members initially voiced concerns that without a scientific background, they would feel out of their depth in understanding research concepts. However, after attending several meetings, all members reported that they had been made to feel at ease through clear and helpful presentations, which were enhanced by interactive role play and workshops:

Beforehand I thought that I might be out of my depth, being a non-academic, but this was not generally the case. I was struck by how a collaborative and consensual framework was established and that we were taken seriously, so that I felt a valued member of the group rather than a token requirement of the project. -Stakeholder

Two group members expressed feeling overwhelmed by the statistics described and believed that it would not be possible for them to understand the analyses despite the presentations and explanations provided. This feeling persisted throughout the 4-year collaboration. An additional member felt that the face-to-face group meetings were well thought out and that the PI’s guidance helped them understand key research concepts:

The workshops contained some really quite challenging concepts, such as statistical analysis…. however, the explanations were very accessible, and I thought that the practicals were quite entertaining, as well as informative. -Stakeholder

The most challenging meeting of the project was, from most of the stakeholders’ viewpoints, the one to discuss the research protocol. There was a lot of new information to read through, which was challenging to all apart from the one stakeholder who had a scientific background. However, the three members who partook in literature screening and data extraction reported that it was a helpful meeting as it helped familiarise them to the project and research process.

The PI expressed concerns around training stakeholders to a level at which they could conceptually understand all the steps of the research and aimed to make the training engaging and didactic. However, over time, the PI’s view was that the group internalised the project more, with members finding their feet more quickly and making more specific suggestions, showing a developing understanding of research terminology and concepts.

### Feelings about stakeholder involvement

All members responded positively to the question regarding how they felt about their involvement as public stakeholders in the project. They described varying reasons for why they rated their experience as positive, including learning a lot about research and the motivation behind it:

I found helping with the research, albeit in a small way, brought the project to life and instilled a better understanding of the why’s and wherefores of systematic reviews. I would have welcomed a bigger role if the opportunity arose. -Stakeholder

The PI described the involvement of stakeholders positively and viewed the group’s impact as important for varying reasons, including in providing helpful feedback, keeping the research relevant to the public, and holding the researchers accountable throughout the long project. The project had been a solitary activity at times so the PI felt accompanied throughout by the stakeholders, as they remained fully involved at all stages and provided a sense of teamwork and support:

There were many great things about involving stakeholders - such as getting lay viewpoints and feedback, getting help with the research itself, meeting new people, learning how to train and engage the public, and having fun during meetings… Also, being accountable to them helped me structure and keep it relevant. I was pleasantly surprised at how refreshing and helpful it can be to share your project with enthusiastic members of the public. – Researcher

The PI expressed some regret concerning a lack of time to involve the stakeholders as much as she wanted and reported that the balance between doing the research and involving stakeholders, due to limited time, had been difficult.

### What went well

All four group members stayed on through the course of the study and reported very positive experiences overall, reporting that they shared a new experience, learned new skills, and made new friends:

Learning new skills, doing something completely different, de-mystifying academia, putting a human face on it, meeting new people from other walks of life…” -Stakeholder

I felt that my favourite thing about the project was being part of a cutting-edge study as to the benefits, or not, of mindfulness. I have never had the opportunity to take part in a stakeholder group and it opened up a whole new area for me with the added blessing of making new friends and providing stimulus regarding a debated subject. Unfortunately, as the experience draws to a close it does leave a void. Overall, it was a real privilege to have been given the opportunity to take part. I would love to be involved in future stakeholder groups. -Stakeholder

All four agreed that meeting new people of different ages and backgrounds was positive and stimulating and that the group blended well and became a friendly and cohesive group.

### What did not go well

The group was aware that the research study, from its outset to conclusion, would take 4 years. However, there were drawbacks to the long breaks between meetings where members felt they had lost some interest in the study and were forgetting details. Two stakeholders suggested some more frequent group meetings:

There were long gaps, during which time I lost interest and forgot where we were at. I wonder if it would have been feasible to organise mini catchups in between where we were required to read through the notes from previous meetings beforehand? -Stakeholder

Another challenge reported by a member was the duration of the meetings as they found that 4-h meetings were difficult, particularly when covering more challenging topics such as statistics. Two members additionally described grappling with multiple documents and PowerPoint to be difficult at times.

The PI would have preferred to hold more regular and frequent meetings. The challenge was to balance the time to carry out the research with the time to meet with stakeholders. It was also deemed unnecessary to convene a meeting when there were limited things to present or discuss with the group. Another point the PI had to consider when planning and running the sessions was that group members had different abilities and understanding, so there was a risk of imbalance which could lead to disinterest by some if it was not as engaging, but frustration for others if too challenging:

At times, the concepts were difficult to grasp so perhaps some became disengaged or struggled to follow the session for a bit, while others could follow me. I tried to accept this as normal; a diverse group means different people will understand different things better. -Researcher

### Felt impact of public stakeholders on research

Stakeholders initially felt their efforts carried little impact throughout the research project; however, by the end of the project, they were able to see some evidence of their involvement on the study outcome:

When we reflected recently, I didn’t feel I’d made very much impact on the research. However, reading the summary of the project, it does sound like the feedback from our group has helped guide decisions in how the research has proceeded. -Stakeholder

Other stakeholders reported feeling that the group’s input and feedback may have prompted the lead PI to do some things differently. One group member described feeling unsure of the group’s impact but hoped that their life experiences and views shared in meetings were able to represent the wider public. It was suggested by members that it would be useful to frequently remind stakeholders about how they are contributing to the project.

While she tried to transmit the usefulness of their contributions informally at each meeting, the PI wondered if one of the reasons that the stakeholders felt that they may not had contributed much to the project was because she did not have a formal and consistent process of giving them detailed feedback on how she had used their suggestions. This lack of formal process was down to the researchers’ lack of time, and the need to prioritise other aspects of the stakeholder involvement with the time available.

### Study outcome

The group had mixed views about the outcomes of the research. Some said they had no real expectations of the outcomes and were hopeful, but in one case, sceptical about its benefits. One participant was disappointed when the conclusions of the first phase of the study were shared:

I initially felt disappointed that the meta-analysis did not show a strong benefit from mindfulness, compared to other interventions, such as exercise. I was expecting a clearer benefit to be seen. However, I think the results resonate with my own experience, in that I find exercise to be more beneficial for my mental wellbeing than meditating. -Stakeholder

### Would they do it again and recommend the role to others?

All four stakeholders said they would be happy to be public stakeholders again, and, with caveats, would recommend the role to others. While positively recommending the role, one stakeholder stated:

…in many ways meta-analysis is an end-game and it may be that individual research projects themselves would be more attractive to some. My advice would be not to be put off if, initially, stakeholders felt out of their depth. This is to be expected if medical research is a completely new experience. A good lead researcher helps allay that feeling by engaging well with the group, simplifying explanations and making meetings stimulating and fun. -Stakeholder

Another member reflected that, ideally, stakeholders should have a general interest in the research subject, and it was important to approach the project with an open mind on the findings—not feeling too invested in arriving at a particular conclusion. It was also important to consider the time pressures involved for stakeholders who have busy working lives.

### Feelings about the group

The group felt the four-member number was adequate, although some pointed out that up to two more participants would, perhaps, have added more diversity and would have been useful in case one or more stakeholders had dropped out along the way.

### Involvement of public stakeholders in future research

With the right amount of time and resources available, the researchers would involve members of the public again in future research. The PI highlights the importance of involving stakeholders as soon as possible, including when putting together the grant application and drafting research protocols. The stakeholder feedback on the research plans and protocol was perceived as being just as essential as their involvement throughout the study so researchers would benefit from early stakeholder involvement. When undertaking an IPD-MA, the PI advises other researchers to involve public stakeholders and to design participation in ways that provide an engaging and interesting experience:

It’s essential to make meta-analysis training as engaging as possible, even if this takes more time and effort. I would also make sure to manage expectations and to remind stakeholders frequently about how they are contributing. -Researcher

## Discussion

Overall, the stakeholders and researchers reported a positive experience of working together throughout this two-stage complex evidence synthesis project. Numerous benefits and some challenges were reported throughout the feedback provided during the 4 years of involvement and within the final questionnaires. Stakeholders reported positively on their involvement, and all said they would participate in a similar project in the future. Positives from the stakeholders’ points of view included learning new skills and making new friends and they generally enjoyed taking part in a research project. The researchers appreciated working with members of the public to help them focus on what matters to the public, found it helpful for stakeholders to carry out research tasks and enjoyed having meetings with an engaged group that brought fresh perspectives to the table.

The challenges stakeholders experienced included having long gaps between meetings, resulting in some waning interest and forgetting project details, and feeling overwhelmed at times with the level of information being described.

Similarly to the stakeholders, the PI would have preferred to have more frequent meetings, but time and funding were limited, so this was not possible. The PI additionally expressed difficulty with getting the teaching level right, particularly as stakeholder backgrounds ranged in experience of research. It was challenging at times to strike the balance between asking stakeholders to be involved and for them to learn research-related skills without overburdening them and making sure that the learning was palatable and engaging.

When looking back at their experience, stakeholders described seeing their impact on the project in hindsight but that this was not felt while the project was being carried out. The general feedback on how things could be improved in future evidence synthesis projects with stakeholders was to involve more stakeholder members, to hold more regular meetings, and to keep these shorter in duration when covering challenging topics such as statistics. It was also suggested that researchers could feed back to the group the influence stakeholders have on the research itself during the project so that they are aware of their impact and feel more useful in their contribution. And finally, despite the efforts of the PI to keep the learning as engaging and not overwhelming as possible, it was a challenge for some to keep up and to use some of the technology, such as Microsoft PowerPoint; hence, the importance of gauging the audience level prior to creating learning material and employing technology that all members are able to use.

The main challenge which came up throughout the course of the project and in the feedback questionnaires was that of overwhelming stakeholders with research terminology and concepts. The PI also found it challenging to have a group with mixed engagement and interest, but it seemed that after a while, stakeholders settled into their roles and became more comfortable with research concepts, although the feeling of uncertainty did persist for two group members. Stakeholders often report enjoying learning new skills [22]; however, researchers should remain vigilant of not burdening stakeholders and turning their experience negative. Generally, stakeholders will partake in projects on a very part-time basis so researchers should consider the challenges stakeholders may face when having to learn new concepts surrounding work, they may have limited interest and time for and to set realistic and achievable learning goals. It is important to understand if stakeholders are unhappy because they may disengage; an alternative could be that those who feel less comfortable with certain concepts are tasked with something they prefer. The group were not expected to apply statistical skills at any point, and rather were tasked with supporting the screening and data extraction which they did pick up more easily and reportedly enjoyed.

Numerous papers have described stakeholders struggling with research concepts and terminology and the stakeholders from Bayliss et al. [7] asked for something like a glossary, which Brütt et al. [5] provided for theirs. It could be that at the start, as Bayliss et al. [7] have done, the group starts with standardised training material and this is slowly developed as the group learns and adapts it to their level of knowledge and skill. An option could be to host a live glossary of terms that people can refer to and when new terms come up, researchers and stakeholders could add terms and definitions to these. However, stakeholders for this project did not feel it was necessary to have a live document. It is fairly evident that different groups have different preferences so researchers should use the initial meetings as an opportunity to ask stakeholder preferences and to co-produce or adapt learning material to their specific needs. This would also be a chance for researchers to understand the group’s level of research knowledge and for stakeholders to communicate their needs.

In general, stakeholders involved in evidence syntheses have reported positive experiences and said they would all do it again [7, 16]. Members were reimbursed for their time, including their preparation for workshops or meetings; this is important as stakeholders should be paid for the experience and feedback provided [23]. This may perhaps also explain parts of the positive feedback received from stakeholders when they are reimbursed appropriately. This shows that factoring in costs for stakeholder activities is important in evidence synthesis project proposals so that stakeholders can be recruited and may be more likely to stay on throughout the entirety of the project.

The challenge associated with questioning the impact of their involvement as stakeholders has been repeatedly brought up by stakeholders of previous evidence synthesis projects [16, 24]. This feeling may be more present for stakeholders taking part in evidence synthesis projects, as opposed to intervention trials, as the outcomes are already pre-selected and reported in published trials which leaves limited room for input, particularly within quantitative evidence synthesis [24, 25]. A suggested solution to this is to involve stakeholders early on in projects, as early as the funding application or protocol registration, if resources allow, to not only benefit the stakeholder-perceived impact and subsequent experience, but also the true impact and subsequent review quality and relevancy. One of the most important impacts stakeholders had in our project was outcome selection, thanks to involving them from inception. Stakeholders in this project mentioned that in hindsight, they were able to perceive their impact but that during the project they questioned it. It seems like it would be beneficial to stakeholders to receive ongoing feedback from researchers on how they are shaping and influencing the project and to see the impact they have.

Additionally, as stakeholders seem to frequently question their effects on the evidence synthesis research project and outcome, and it is not often formally measured [26], it appears essential for researchers to have a measure of impact developed. A formal measure for stakeholder impact in evidence synthesis projects would be helpful for researchers, for funding bodies, and for stakeholders themselves [26]. Furthermore, there is a lack of collecting data and reporting on stakeholder experiences. The more researchers report on the actual experience of stakeholders, rather than reporting on their behalf, the more in-depth feedback we will have to help guide and improve the experience for future stakeholders.

The challenges reported by stakeholders and researchers in this paper pertaining to research tasks and logistics, such as payments and meeting attendance, are transferrable across the majority of evidence syntheses projects; however, researchers should be aware of unique challenges reflecting their SR PICO (Population, Intervention, Comparator, Outcome) they may encounter. Our stakeholder group was non-clinical and the SR looking at mental health promotion mindfulness interventions, however, researchers working with patient stakeholder groups (e.g. involving stakeholders with depression when synthesising exercise interventions in depression) should plan ahead for potential challenges catering to different support needs. When including interventions which may be slightly more technical in the terminology used in associated publications (e.g. examining underlying mechanisms of pharmaceutical treatments), researchers may need to develop more in-depth training for stakeholder members and dedicate more time to making the project information accessible.

Stakeholders made a clear difference in the research process by carrying out record screening and data extraction, which reduced the researcher workload. They additionally were involved in the co-production of a project film which has been shared publicly. Their involvement made the film content and language accessible, which may have increased the impact of the study output by reaching a wider audience.

Finally, their help in planning out the SR and MAs, including outcome, control group, and subgroup analysis selection, has potentially improved the generalisability and applicability of research findings due to being guided by those with experience of the intervention and for whom the findings would be relevant to. The feedback from the stakeholders also led to the PI seeking further clarifications from a sociologist to best strategise the ways in which the countries in which the interventions were carried out could be grouped for subgroup analysis; this led to a more appropriate cultural subgroup analysis.

There are currently limited guidelines for researchers to involve public and patient stakeholders in their evidence synthesis projects. As the number of evidence synthesis projects continues to grow [27] and research funding bodies emphasise the need for public and patient involvement in projects [28, 29] it is important to have these guidelines in place to ensure a positive experience for all involved parties. To develop guidelines, it is essential to have data on the impact and experiences from those involved, which is what we hope to contribute herein.

Moving forward, it is anticipated that future research will involve stakeholders at increasing rates in evidence synthesis projects and that researchers will endeavour to enhance involvement impact and experience through more in-depth assessments and reporting on stakeholder involvement. Measuring the impact of stakeholder involvement during a research project may become more established and evidence-based, as funders may become interested in measuring the impact in the longer term to justify costs of stakeholder involvement. To measure longer-term impact, it could be useful to compare the impact of projects which have been carried out with and without stakeholder involvement. As some of the justifications for stakeholder involvement include higher impact due to higher ecological validity and applicability (e.g. higher intervention uptake), it could be beneficial to examine the use of the evidence from projects with versus without stakeholder involvement.

It would also be useful for researchers and funders to see the influence that stakeholder involvement has on the wider general population understanding of and interest in science and healthcare research. Important to note for researchers considering stakeholder involvement in future evidence synthesis projects is the rapid rise of popularity of data-driven healthcare [30], use of artificial intelligence (AI) in evidence syntheses [31], and living systematic reviews (LSRs) [32] as these introduce unique considerations when planning and carrying out work with stakeholders. For example, involving AI and public stakeholders in screening activities simultaneously in a project may require more thorough training for stakeholders to guarantee screening accuracy so that the data can be used to train the AI model for screening. Finally, reporting on stakeholder involvement in SRs may become more mainstream which in turn may provide further evidence to develop quality guidelines for involving stakeholder members.

### Limitations

We repeatedly asked group members to be as honest as possible in their feedback, but there remains the chance that responses deviate from actual experience to prevent giving negative feedback to the researchers. Unfortunately, we were unable to measure and report on the long-term impact of stakeholder involvement in an evidence synthesis project. Additionally, for approximately 18 months, in response to the Covid-19 pandemic regulations in the UK, we met online to carry out meetings and workshops. Effort was placed in maintaining the same dynamic as in-person training and meetings; however, as participants in Bayliss et al. [7] report, face-to-face training was seen as more beneficial and as a preferred method of meeting so this may have impacted our stakeholder experience.

## Conclusion

To our knowledge, this is the first IPD-MA project with public stakeholder involvement. A limited number of IPD-MA projects have involved patient stakeholders and there has been some public stakeholder involvement in AD-MAs. Overall, involving a public stakeholder group in the SR process was a positive experience for the researchers and stakeholders, with minor caveats. However, these caveats, such as challenging training concepts or a sense of low impact by the stakeholders, could be better managed by the researchers if there were guidelines and established training material available. Successfully involving the public in complex evidence synthesis projects requires a significant time, skill, and resource investment that needs to be factored in from project inception.

## Data Availability

Data sharing is not applicable to this article as no datasets were generated or analysed during the current study.

## Abbreviations

PPI: Patient and public involvement
SR: Systematic review
MA: Meta-analysis
AD-MA: Aggregate data meta-analysis
IPD-MA: Individual participant data meta-analysis
RCT: Randomised controlled trial
PI: Principal investigator
MBSR: Mindfulness-based stress reduction
AI: Artificial intelligence
LSR: Living systematic review
